# The Role of Ceramides in Insulin Resistance

**DOI:** 10.3389/fendo.2019.00577

**Published:** 2019-08-21

**Authors:** Emilia Sokolowska, Agnieszka Blachnio-Zabielska

**Affiliations:** Department of Hygiene, Epidemiology and Metabolic Disorders, Medical University of Bialystok, Bialystok, Poland

**Keywords:** insulin resistance, obesity, ceramide, inflammation, diabetes, therapy

## Abstract

Resistance to insulin is a pathophysiological state related to the decreased response of peripheral tissues to the insulin action, hyperinsulinemia and raised blood glucose levels caused by increased hepatic glucose outflow. All the above precede the onset of full-blown type 2 diabetes. According to the World Health Organization (WHO), in 2016 more than 1.9 billion people over 18 years of age were overweight and about 600 million were obese. Currently, the primary hypothesis explaining the probability of occurrence of insulin resistance assigns a fundamental role of lipids accumulation in adipocytes or nonadipose tissue (muscle, liver) and the locally developing chronic inflammation caused by adipocytes hypertrophy. However, the major molecular pathways are unknown. The sphingolipid ceramide is the main culprit that combines a plethora of nutrients (e.g., saturated fatty acids) and inflammatory cytokines (e.g., TNFα) to the progression of insulin resistance. The accumulation of sphingolipid ceramide in tissues of obese humans, rodents and Western-diet non-human primates is in line with diabetes, hypertension, cardiac failure or atherosclerosis. In hypertrophied adipose tissue, after adipocytes excel their storage capacity, neutral lipids begin to accumulate in nonadipose tissues, inducing organ dysfunction. Furthermore, obesity is closely related to the development of chronic inflammation and the release of cytokines directly from adipocytes or from macrophages that infiltrate adipose tissue. Enzymes taking part in ceramide metabolism are potential therapeutic targets to manipulate sphingolipids content in tissues, either by inhibition of their synthesis or through stimulation of ceramides degradation. In this review, we will evaluate the mechanisms responsible for the development of insulin resistance and possible therapeutic perspectives.

## Introduction

Consumption of a high-fat diet is associated with the accumulation of lipids in skeletal muscles, which leads to many disorders, like obesity, insulin resistance, type 2 diabetes mellitus (T2DM), and metabolic syndrome (1). Obesity is the most common trigger for the development of insulin resistance in skeletal muscles and the liver. Both are an integral part of metabolic syndrome, together with glucose intolerance, hypertriglyceridemia, low HDL cholesterol, hypertension, atherosclerosis, and impaired fibrinolytic capacity (2). Metabolic syndrome significantly increases the risk of cardiovascular system diseases. WHO global report of diabetes from 2016 estimates that more than 422 million adults were living with diabetes in 2014, half of them in Asia (3). In 2016 there were 1.6 million deaths worldwide directly caused by diabetes. Statistical data shows that in 2012, diabetes was the fifth most common cause of premature death among women.

**Figure 1:**
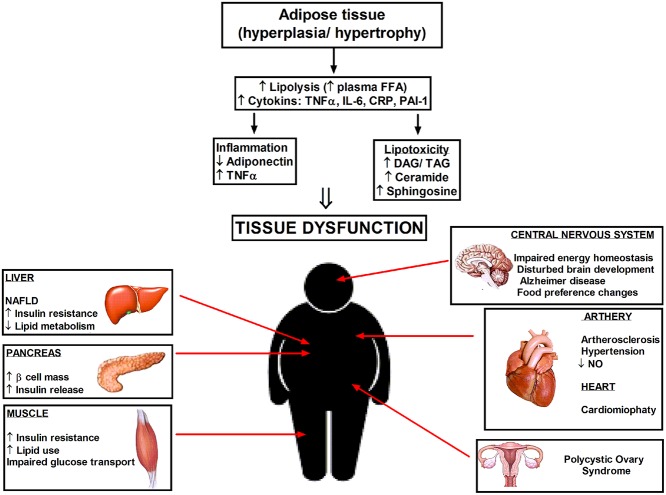
Hyperplasia and hypertrophy of adipose tissue cause systemic inflammation and lipotoxicity due to elevated cytokines and accumulation of lipid metabolites in the tissues not suited for fat storage. Increased ceramide level in enlarged adipocytes elicit the pathophysiological disturbances in different tissues and organs. DAG, diacylglycerol; TAG, triacylglycerol; NAFLD, nonalcoholic fatty liver disease.

Insulin resistance occurs in almost 20–25% of the human population and is defined as a decreased response of the peripheral tissues to insulin action, and consequently impairment in postprandial nutrient storage mainly in skeletal muscle and in the liver. At this stage, pancreatic islets are not yet damaged and respond to elevated blood glucose levels by insulin oversecretion, resulting in their hypertrophy and necrosis (4). On the other hand, as a consequence of continuously maintaining high concentrations of insulin, peripheral tissues are becoming resistant to this hormone. Insulin resistance and weight gain are contributing factors to stroke, nonalcoholic fatty liver disease (NAFLD), asthma, cancer, polycystic ovary syndrome, Alzheimer's disease, hypertension or atherosclerosis (Figure 1) (5). The insulin signaling system is complex and manifold. However, genetic approaches provide valuable insight into certain early-onset forms of diabetes but fail to clarify insulin resistance (6). Ceramides, reactive oxygen species, diacylglycerols, branched chain amino acids, short-chain acylcarnitines, and other metabolites have all been implicated as antagonists of insulin action. Herein we will review suggested mechanisms responsible for the onset of insulin resistance and perspectives to the treatment of patients at risk of diabetes.

## Insulin-Dependent Glucose and Lipid Metabolism

Insulin is the principal anabolic hormone secreted by pancreatic β-cells, which stimulates uptake and storage of glucose and other nutrients in muscle and fat. It modulates post-meal balance of carbohydrates, lipids, and proteins by increasing lipogenesis, glycogen, and protein synthesis, and suppressing glucose production, and its release from the liver. Insulin resistance causes profound dysregulation of these processes and is related with inhibition of the insulin signal transduction downstream to the insulin receptor and phosphorylation of the insulin receptor substrates proteins (IRS 1–4), in particular, IRS-1 (5, 6). In several pathological conditions such as T2DM and metabolic syndrome, the physiological dose of insulin cannot obtain anabolic responses in peripheral tissues (2).

Insulin acts via its transmembrane, heterotetrameric receptor which belongs to a large family of tyrosine kinase receptors. The activated insulin receptor phosphorylates endogenous insulin receptor substrates family proteins that activate intracellular effector enzymes, like the lipid phosphatidylinositol 3-kinase (PI-3K) (7), which stimulates the Akt kinase/protein B (PKB). PKB regulates cell proliferation and survival, but it is thought to contribute to several cellular effects including the stimulation of glucose transport, nutrient metabolism, cell growth, or transcriptional regulation (8). Therefore, in muscle and fat tissue, Akt/PKB stimulates the transfer of insulin-dependent glucose transporter (Glut 4) from the depot inside the cell to the cell membrane. Thus, it facilitates the inflow of glucose to the muscles (9). Akt/PKB also promotes system an amino acid transport (10), and vasodilatation by activation nitric oxide synthase (11). Additionally, Akt/PKB stimulates in liver and muscles glycogen synthase kinase 3b (GSK3b) (12), and in muscles and in fat tissue induces protein synthesis (13). In fat tissue, Akt/PKB promotes expression and activity of fatty acid synthase, which is linked to visceral fat accumulation by catalyzing multiple reactions in the conversion of malonyl-CoA and acetyl-CoA to long-chain fatty acids (14).

## Hypotheses of Insulin Resistance

**Figure 2:**
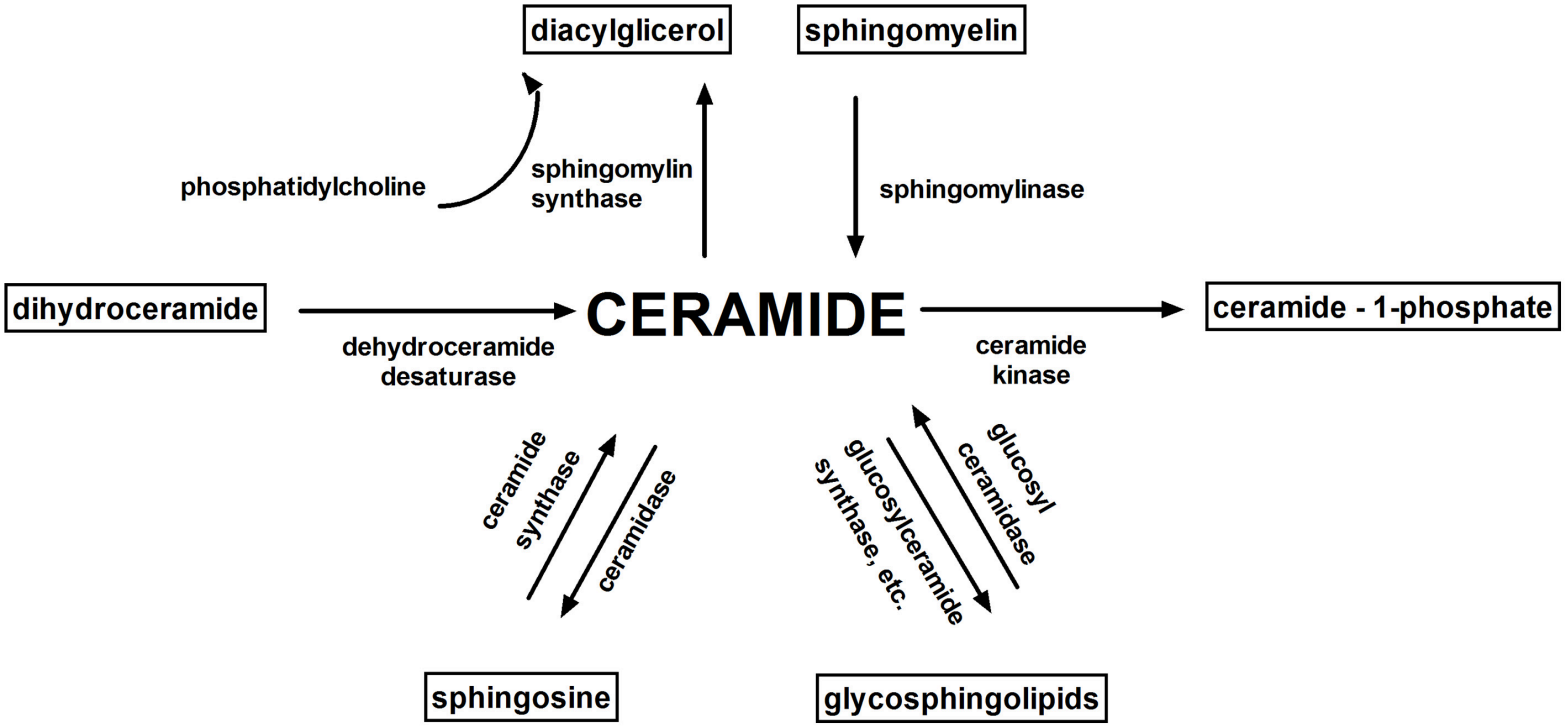
Sphingolipid metabolism. Ceramide is a key molecule in sphingolipid metabolism. The main bioactive sphingolipids: ceramide, sphingosine, sphingosine 1-phosphate, ceramide-1-phosphate play an important role in cell signaling.

Accumulation of intramuscular lipids, in particular: diacylglycerols (DAG), ceramide (Cer) and long-chain acyl-CoAs (LCACoA) (15–18) (which are substrates in the *de novo* synthesis of both ceramide and DAG) are proposed to be involved in the induction of insulin resistance. Bioactive sphingolipids like ceramide, sphingosine (Sph), and sphingosine 1-phosphate (S1P) combine overnutrition, inflammation, and metabolic dysregulation (19) (Figure 2).

Nuclear magnetic resonance spectroscopy has demonstrated a relation among intramyocellular irregularity of lipid metabolism and triglyceride level with whole-body insulin resistance in overweight patients and those with type 2 diabetes (20). Excess of fatty acids is stored in adipocytes to support energy during fasting periods. After exceeding the buffering capacity of adipose tissue, neutral lipids accumulate in nonadipose tissues such as liver, heart, pancreas, and skeletal muscle, inducing organ dysfunction called lipotoxicity (Figure 1). This anomaly increases the probability of the onset of two molecular pathogenesis responsible for the persistent hyperglycemia observed in T2DM like the progressive decrease in function and the mass of pancreatic β-cells (21). Muscle tissue adjusts to increased clearance of free fatty acids (FFA) from the circulation and lipids begin to accumulate in skeletal muscle. It is likely that a significant uptake of FFA by muscle may be the result of increased mRNA expression for the CD36/FAT transporter and the acyl-CoA synthetase. Furthermore, intramyocellular lipids levels were more effective in the prognosis of insulin resistance than were other predictors of obesity (e.g., waist-to-hip ratio, body mass index (BMI), and body fat percentage) (22, 23). Moreover, LCACoA through inhibition of hexokinase (24), pyruvate dehydrogenase and glycogen synthase activity (25), decrease the insulin signaling. LCACoA also disrupt muscle glucose utilization by stimulation of PKC isoenzymes which activate serine/threonine phosphorylation of the insulin receptor or insulin receptor substrate 1 (IRS-1) (26). Moreover, palmitoyl-CoA inhibits mitochondrial adenine nucleotide translocator, resulting in the greater formation of reactive oxygen species (27). It was shown that muscle insulin resistance arises from impaired mitochondrial uptake and oxidation of fatty acids (28). Excessive supply of nutrients circulating in the blood and their uptake by the cells increase the rate of cell metabolism (described as metabolic overload), impair the activity of mitochondrial and endoplasmic reticulum-associated enzymes, decrease the accumulation of acetyl coenzyme A in cells, and inhibit glycolysis by suppressing the activity of key enzymes in the Krebs cycle (29).

The short-chain fatty acids, same as glucose, enhance insulin secretion, while chronic exposure of pancreatic islet to high concentrations of FFA lead to desensitization and inhibition of insulin release (30) as well as lower insulin gene expression (31). Disproportionate accumulation of FFA in cells of overweight people impairs signaling pathways regulated by diacylglycerol (DAG). Recently, more attention is being paid on DAG because of their suggested role in the initiation of insulin resistance in muscle and liver (32). Ceramides have a detrimental effect on pancreatic β cells, where they activate the stress-induced apoptotic pathway (i.e., cytochrome C release and free radicals production) (33).

The results of dietetic, epidemiological, and animal studies clearly show that the consumption of saturated fats (SAT) decrease sensitivity to insulin (34). Among SAT, palmitate is remarkably harmful, as initiate accumulation of ceramide, which impairs insulin-dependent activation and signaling of PKB/Akt [downstream mediator of the insulin receptor (IR)] (35) to relevant endpoints, such as glucose transport by: simplify signaling pathways initiated by inflammatory cytokines (TNFα) (36), activation of protein phosphatase 2A or atypical protein kinase C isoform (PKCζ) (35). Infusion of a triglyceride emulsion into humans elevates DAG amount in muscles, but not ceramides (37, 38). Data from our group have shown that diet rich in SAT, in contrast to a diet rich in polyunsaturated fatty acids (PUFA), leads to an elevation in ceramide (1), pointing this sphingolipid as a strong candidate combining lipid oversupply to the antagonism of insulin signaling (35). Research conducted by Pinel et al. in C2C12 myotubes indicate that n-3-PUFA may display their beneficial metabolic effects on skeletal muscle and enhance insulin sensitivity by improved metabolism of fatty acids in skeletal muscle cells, in particular through their ability to increase mitochondrial β-oxidation (39). Bikman et al. showed that monounsaturated fatty acid oleate prevents insulin resistance by inhibition of the ceramides synthesis in the presence of palmitate (40). Athletes also become insulin resistant after administration an unsaturated lipid emulsion. However, thanks to their better metabolic flexibility, the increase in fatty acid oxidation compensate the decreased glucose oxidation. It seems that the accumulation of FFA in tissues not adapted to their storage is not directly responsible for the development of insulin resistance (41). Reduced tissue sensitivity to insulin is also associated with disturbances in the functioning of nuclear receptors PPAR family and the changes in the secretion of adipokines (leptin, adiponectin, and resistin) and proinflammatory cytokines (IL-6, TNFa) (42). In fat pad of obese rodents and humans, overexpression of TNFα promotes insulin resistance through increased serine phosphorylation of the IRS-1 receptor and decreased insulin receptor kinase activity (43). It was also shown that intraperitoneal injection of TNFα into C57BL/6J mice upregulates the activity of neutral and acidic sphingomyelinases and therefore produces ceramide in excess (44).

## Ceramides and Insulin Resistance

Ceramides are important bioactive lipids belonging to the sphingolipid family produced from a fatty acid and sphingosine or by sphingomyelin hydrolysis (21). Ceramides in biological membranes are part of the membrane microdomains, known as lipid rafts, which stabilize the cell membrane structure and modulate the distribution of receptors and signaling molecules. Additionally, ceramides affect cell signaling pathways that mediate growth, proliferation, motility, adhesion, differentiation, senescence, growth arrest, apoptosis (33, 36, 45). These compounds regulate the activity of many enzymes like kinases or phosphatases but also can alter the activity of transcription factors (46, 47).

### Ceramide Synthesis and Degradation

The rate of ceramides generation depends mainly on the availability of long-chain saturated fatty acids, which participate in the novo ceramide synthesis within the endoplasmic reticulum (48). Ceramides vary in acyl-chain lengths from C14:0 to C30:0. At the first, rate-limiting step of the *de novo* pathway, serine palmitoyltransferase (SPT) initiates the condensation of serine and palmitoyl-CoA to produce 3-ketosphinganine. Further reactions lead to the formation of sphinganine which is acylated to dihydroceramide by six ceramide synthase enzymes (CerS1–6), and then to ceramide through dehydroceremide desaturase. Among various acyl-CoAs, palmitoyl-CoA is a highly specific substrate of mammalian SPT *in vitro* and consequently, in mammals, the chain length of the sphingoid bases is mainly C18 (48, 49). Recent studies revealed that exogenous fatty acids, cytokines (i.e., IL-1), and even UVB radiation elevate ceramide generation by upregulating the expression of SPT (48, 50).

Ceramide may also be formed by hydrolysis of sphingomyelin by the neutral and acid sphingomyelinases (nSMase and aSMase), which is a stress-activated pathway for ceramide synthesis. These enzymes break down sphingomyelin into ceramide and phosphocholine and are upregulated in response to TNF-α (51), Fas ligand (52), TLR4 activation (53), or oxidative stress (54).

**Figure 3:**
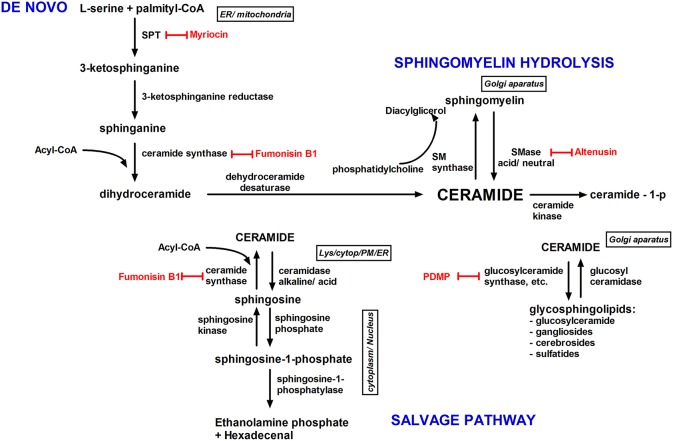
Ceramide biosynthesis pathways and their subcellular localization. Ceramide is synthesized by three pathways; the *de novo* pathway; the sphingomyelin pathway; and the salvage/recycling pathway. The *de novo* synthesis pathway (endoplasmic reticulum), begins by the condensation of serine with palmitoyl-CoA by serine palmitoyltransferase into dihydrosphingosine. Next, dihydrosphingosine is converted into ceramide. In the salvage pathway, sphingosine is metabolized into ceramide by ceramide synthase, and glucosylceramide is degraded into ceramide by glucosylceramidase. The recycling pathway is accountable for the cycling among ceramide and complex sphingolipids. In the sphingomyelin hydrolysis pathway, plasma membrane sphingomyelin is hydrolyzed into ceramide by sphingomyelinase. Inhibitors of ceramides metabolism are shown in red. Cytopl, cytoplasm; ER, endoplasmic reticulum; lys, lysosome; PM, plasma membrane; SMase, sphingomyelinase; SM synthase, sphingomyelin synthase; SPT, serine palmitoyltransferase.

Ceramide can be phosphorylated (by ceramide kinase), rapidly deacylated (by ceramidase, CDase: acid, neutral, and alkaline), or glucosylated (by glucosylceramide synthase) (55). The enzyme sphingomyelin synthase (SMS) transfer a phosphocholine group from phosphatidylcholine to ceramide, whereby sphingomyelin (SM) is reformed (56) (Figure 3).

### Does Tissue Ceramide Level Effectively Predict the Insulin Resistance?

Human studies indicate a connection between ceramides and insulin resistance. The accumulation of ceramides can increase in tissues due to excessive supply of either saturated or unsaturated fatty acids, most likely as a result of sphingolipid recycling or the salvage pathway activity. For example, elevations in muscle ceramides were reported in individuals with general or abdominal obesity in association with muscle insulin resistance (57, 58). Correlations between liver ceramides and hepatic insulin resistance and between adipose ceramides and fatty liver disease are also reported (59). However, the accumulation of fatty acids does not always correlate positively with the decrease in tissue sensitivity to insulin.

Caloric restriction diet, exercise training or bariatric surgery reduce muscle ceramide levels and enhance muscle insulin sensitivity, while levels of DAG in skeletal muscle in endurance-trained athletes were higher (57, 60, 61). This phenomenon is known as “the athlete's paradox” (41) and is in line with studies which have shown that DAG maintain a certain balance in the body, simultaneously preventing the accumulation of more reactive species of lipid metabolites in muscles (62).

Ceramides antagonize insulin signaling by inhibiting transmission of signals through phosphatidylinositol-3 kinase (PI3K) and blocking activation of the anabolic enzyme Akt/PKB (63). Thereby, ceramides interfere with glucose uptake and impair storage of nutrients such as glycogen or triglyceride (21), activate protein phosphatase 2A (PP2A) (15, 35) and activate proinflammatory cytokines. These sphingolipids also disrupt lipid metabolism, particularly in the liver, by inhibiting oxidation and stimulating fatty acid uptake (21). Moreover, ceramides enhance cell death by stimulation of caspase, protein kinase C, serine/threonine protein phosphatase (PP1), and cathepsin D activity (33, 45).

Plasma ceramides are higher in obese children (64), diabetic adults (65), or in non-human primates fed a Western diet (66). It was shown that decrease in plasma ceramides during pioglitazone treatment (67), inhibition of ceramide synthesis (i.e., myriocin, fumonisin B1) or stimulation of ceramide degradation (i.e., acid ceramidase overexpression) improves insulin signaling (21). Furthermore, the addition of SPT inhibitors (myriocin or L-cycloserine) to muscle cell lines L6 (rat normal skeletal muscle myoblasts) significantly preserved both PKB activity and insulin-stimulated glucose transport (68). Manukyan et al. have shown, that C14, C16, and C24 ceramides produced by sphingosine acylation in response to enhanced palmitate levels contribute to the disturbance of insulin secretion (69).

Taking all into account, a number of studies carried on humans, rodents and cell cultures indicate the undeniable participation of tissue and plasma ceramides in obesity and the development of insulin resistance.

#### Skeletal Muscle

Sphingolipids were identified in various tissues in all higher organisms. The vast majority of the works related to the problem of obesity and insulin resistance focused on the metabolism of sphingolipids in skeletal muscles (1, 18, 41, 70, 71), which constitute 40% of the human body and are responsible for 70–80% of whole body insulin-stimulated glucose uptake. Furthermore, enhanced the intracellular level of ceramides in the muscles of insulin-resistant, overweight humans (58, 70) directly inhibit insulin-stimulated glucose uptake in L6 myotubes, reduce Glut4 translocation and is followed by a reduction in levels of activated Akt/PKB through different mechanisms (72). First, ceramides stimulate activation of atypical PKC isoforms (PKCζ/λ), which favors their combination with PKB/Akt and further prevents the activation of PKB/Akt in response to insulin (35, 46, 73). Moreover, ceramides indirectly suppress the PKB/Akt activity by stimulation of PP2A (73).

The level of ceramides has also been measured in insulin-sensitive tissues. Patients with type 2 diabetes, almost double the level of ceramides in muscle cells (18, 57). However, Skovbro et al. demonstrated that the amount of ceramides in human skeletal muscle is not the principal determinant of muscle sensitivity to insulin (74). In a similar study, Straczkowski et al. after administration of a lipid infusion in humans, found a significant negative relationship between the ceramide level in skeletal muscle and the degree of insulin resistance (71). These conclusions assume that the accumulation of lipids in nonadipose tissues may block glucose disposal. Interestingly, in contrary to muscle and adipose tissue, *de novo* synthesized ceramides are not stored in the liver. Watt et al. observed a liver removal of newly synthesized ceramides following the infusion of lipids in healthy volunteers, which prevent the accumulation of ceramides in the body (75). We have shown that elevated plasma FFA increase the activity of ceramide metabolism enzymes, especially SPT, and promote *de novo* ceramide synthesis (76). Also, Adams et al. showed that ceramide content correlates with the plasma FFA concentration (70). In diabetes, there was an increase in activity of nSMase (an enzyme involved in the generation of ceramide) and the inhibition of aSMase. Our study showed that elevated plasma FFA concentration in nondiabetic animals stimulate the nCDase activity in the soleus and red section of the gastrocnemius. The sphingosine elevation can be a result of increased activity of sphingosine-1-phosphate (S1P) phosphatase or decreased activity of sphingosine kinase, what could explain the fact, why the level of S1P does not change although the sphingosine content increased (76).

#### Adipose Tissue

Adipose tissue is the most effective energy storage organ that manages whole-body energy homeostasis and acts as a decoy for fatty acids plethora coming from nutrients (77). Several studies prove the presence of ceramide in adipose tissue (44, 59). In 3T3-L1 and brown adipocytes, enhanced ceramide can deregulate both insulin-stimulated Glut4 expression and glucose uptake, may also mediate the effect of TNFα on Glut4 mRNA amount. In brown adipocytes TNFα influence on insulin action through *de novo* ceramide synthesis, while in 3T3-L1 adipocytes the Glut4 expression is inhibited by ceramide produced via sphingomyelin hydrolysis (78). In leptin-deficient, genetically obese (ob/ob) mice, the hyperinsulinemia and increased TNFα associated with obesity lead to the elevated expression of three prominent enzymes taking part in ceramide generation in adipose tissue: nSMase, aSMase, and SPT (44). In adipocytes of obese people, the greater ceramide level is probably the result of the increased activity of enzymes reliable for ceramide production (SPT, nSMase). In obese females, the increased activity of nCDases in adipose tissue is a compensatory mechanism that reduces the accumulation of ceramide in adipocytes. Kim et al. suggest that adipocyte hypertrophy is a leading cause of insulin resistance in early obesity, regardless of inflammatory responses (79).

##### Anti-insulin resistance adiponectin action via ceramide degradation

Adiponectin, an adipocyte-secretory factor poses autocrine and paracrine functions. It was shown that adiponectin exerts a variety of beneficial systemic action by lowering triglyceride content in muscle and liver in obese mice and enhance insulin sensitivity (80). Furthermore, only when adiponectin and leptin were administered simultaneously in physiological concentrations the resistance to insulin in a mouse model of lipoatrophic diabetes was reversed. Declined adiponectin level causes a reduced response to the insulin action what indicates that the adiponectin may become a way of treatment of insulin resistance and T2DM (81). For clinicians, the level of adiponectin is an attractive parameter determining the qualitative status of adipose tissue and the general health of the patient. Holland et al. showed that increased concentration of circulating adiponectin in mice reduces ceramides in different tissues (82). Adiponectin increases the activity of ceramidase linked with its two receptors, AdipoR1 and AdipoR2 and enhances ceramide catabolism to from sphingosine. In the next step, through phosphorylation is formed sphingosine-1-phosphate (S1P), a major bioactive antiapoptotic sphingolipid metabolite (83) (Figure 3).

## Inflammation and Insulin Resistance

**Figure 4:**
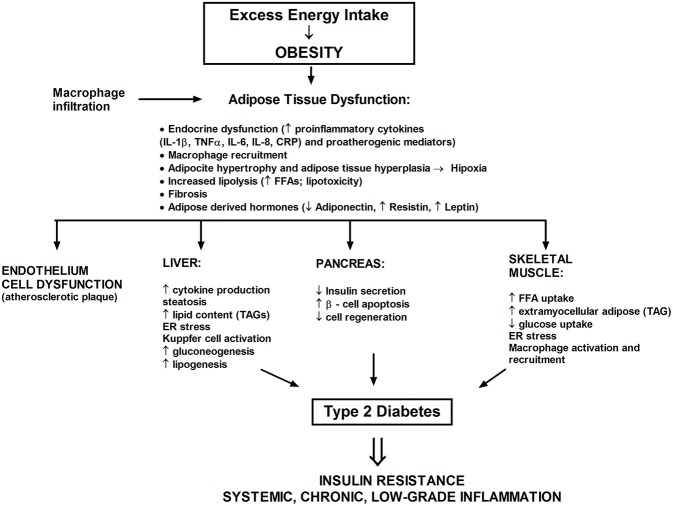
The excess saturated fatty acids dysregulate a white adipose tissue homeostasis and lead to the development of metabolic stress. A chronic state of inflammation cause infiltration of adipose tissue by macrophages and local insulin resistance. Adipose tissue also serves as an endocrine organ whereby proinflammatory mediators reach the nonadipose tissue such as liver, muscle, or pancreas.

Obesity correlates with as a state of persistent low level inflammation (84), which likely cooperate to ceramide cumulation (44, 65, 80) (Figure 4). A broad spectrum of investigations have related insulin resistance with systemic inflammation and oxidative stress but also with dysregulated lipid metabolism and elevated ceramide production. In obese, Caucasian patients with T2DM blood levels of proinflammatory cytokines (such as IL-1, IL-6, and TNFα) are enhanced. The significant rise of serum sialic acid (an indicator of the acute-phase reaction), α-1 acid glycoprotein, IL-6 among three groups were also observed, as follows: the lowest content in nondiabetic patients, middle content in a T2DM group without metabolic syndrome, and the highest content in patients with metabolic syndrome (85). The TNFα ligand-free mice or lacking the p55 TNF receptor were secured from insulin resistance induced by adiposity (86). Silencing of genes encoding TNF, IL-1α or one of the stress-induced kinases—JNK-1, increased the sensitivity of peripheral tissues to insulin (84). Also, the chemokine monocyte chemotactic protein-1 (MCP-1) decrease adipocyte insulin sensitivity (87).

In obesity, adipose tissue grows excessively (hypertrophy) and adipocyte number increases (hyperplasia) (79). Distribution of fat tissue is also very significant. Visceral adipose tissue, compared to the subcutaneous, is less sensitive to the antilipolytic action of insulin and therefore releases more FFA (88). Hypertrophy contributes to hypoxia of adipocytes, which activates the HIF-1 gene, leads to cell stress, modulates the population of immune cells (i.e., macrophages), activates the cells death, stress-induced kinases (JNK, PKCθ) and transcription factor NFκB (89, 90). Furthermore, NFkB in adipocytes increases expression of genes encoding proinflammatory cytokines, adipokines, and a chemoattractant (e.g., MCP-1), what leads to monocyte recruitment to the adipose tissue where they differentiate in macrophages and further secrete proinflammatory cytokines (87, 89, 90). Thereby, a significant disturbance of insulin cellular signal transmission can occur in the adipocytes, ultimately resulting in enormous adipose tissue lipolysis, cell death, and systemic insulin resistance. Moreover, excessive growth of fat tissue, disrupt the production of adipokines, and so the leptin level increases and adiponectin concentration decreases, which is directly connected with the progression of insulin resistance and the onset of full-blown T2DM (84). Simultaneously with the raised inflammation state, M1 macrophages migrate intensively into adipose tissue. In obese patients, more than 50% of the mass of adipose tissue may contain activated proinflammatory M1 macrophages in contrary to slim individuals, whose adipose tissue contain <10% of M2 macrophages. All of the above is responsible for phosphorylation of IRS-1 protein on serine residues located at positions 302 and 307, what prevents the phosphorylation of the insulin receptor on tyrosine residues and consequently leads to inhibition of the insulin signal transduction (26, 46). Increased lipolysis of WAT results in the release of a large amount of FFA, which leads to resistance to insulin action in skeletal muscle and liver (20, 22, 75, 76). Therefore, it is possible that FFA are a relevant link between long-term fat pad inflammation and systemic insulin resistance. Researchers assumed that inflammation in obese patients decrease the insulin sensitivity, as that adipose tissue is a source of proinflammatory cytokines from adipocytes and macrophages that infiltrate fat tissue (90, 91).

## Therapeutic Perspectives of Insulin Resistance Prevention

One of the therapeutic possibilities in the prevention of insulin resistance is selectively increased metabolism of fatty acids in adipocytes by elevated activity of acetyl coenzyme carboxylase A (ACC) or kinase activated by AMP (AMPK). Several studies concern influence on inflammatory factors and their receptors. Researchers are trying to: (1) block the TNF receptors using antibodies (90); (2) use of anti-inflammatory properties of activators of PPAR family receptors (67); (3) use of anti-inflammatory properties of statins (so far clinical studies failed to demonstrate their impact on insulin resistance); (4) use non-acetylated salicylates which efficiency is checked in clinical trials in patients with T2DM (e.g., Trilisate, Disalcid) (92). Salicylates are useful in the treatment of inflammatory states such as rheumatic fever and rheumatoid arthritis, but when administered in high doses lower blood glucose concentrations (92). Furthermore, salicylate-based inhibitors or declined IKKβ expression reduce signaling through the IKKβ pathway, the main pathway in tissue inflammation, which leads to the improvement of insulin sensitivity *in vivo* (92, 93). Systemic insulin sensitivity is also enhanced in JNK1 mice deficient, another pivotal mediator of inflammatory reactions (92, 94).

Many of sphingolipids present in mammalian cells play essential roles in tissue function (95), yet, increased ceramide content in cells correlates with elevated inflammation and insulin resistance (18, 21, 51, 70). Inhibition of ceramide generation prevents lipid- and diet-induced-insulin resistance, or hepatic steatosis (34, 96, 97). Thus, prevention of these lipids harmful actions appears to be an attractive therapeutic approach. Two strategies are taking into account: (1) acting on PKCζ/λ or PP2A that are ceramide downstream signaling targets (15, 35, 45) or (2) modulating ceramide content. However, the only second possibility seems to be interesting. Furthermore, PKCζ/λ and PP2A are relevant components of numerous pathways, thus it would be problematic to precise influence their function in muscle cells without causing undesired actions. Nevertheless, global inhibition of ceramide production could also be harmful.

### Serine Palmitoyltransferase (SPT)

Mammalian SPT is a heterodimer, which belongs to the family of pyridoxal 5′-phosphate-dependent enzymes, present in the endoplasmic reticulum (ER), and is primarily composed of two distinct subunits, Sptlc1, and Sptlc2 (49, 95). SPT is necessary for the *de novo* production of ceramide from palmitate and is a possible central player in suppressing lipid-induced insulin resistance. SPT activity is inhibited by serine analogs such as cycloserine and by the antifungal agent myriocin (Figure 2) (49). Physiologically, the intracellular amount of 3-ketosphinganine, sphinganine, and ceramide generated through different sphingolipids metabolism pathways remain at rather low levels (98). However, the increased availability of circulating saturated fatty acids, particularly palmitate, significantly enhance ceramide generation, which cellular cumulation is proapoptotic (99), as well as impairs insulin action in skeletal muscle and adipose tissue (21). Holland (34) suggested that SPT may be the relevant molecular goal for antidiabetic therapies while the myriocin used in rodents improves some of the metabolic disturbances correlated with glucocorticoid-, saturated fat- and obesity-induced insulin resistance. Inhibition of Sptlc2 in subcutaneous WAT prevented HFD-induced adipocyte hypertrophy and was adequate to alter whole-body energy expenditure, as a consequence of decreased adiposity (100).

### Dihydroceramide Synthases (CerSs)

**Figure 5:**
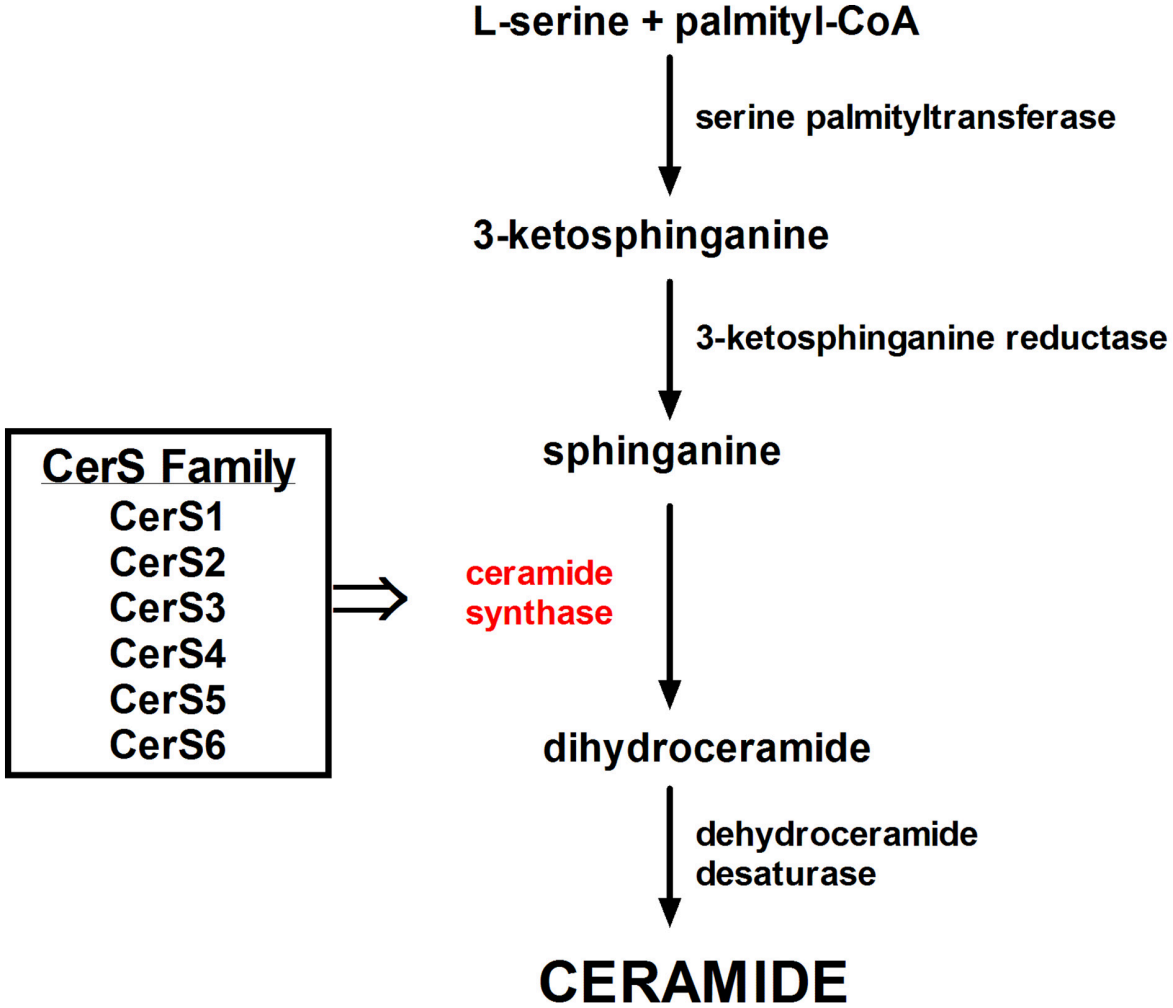
The family of Ceramide Synthases acylates sphinganine to form dihydroceramide.

A family of six enzymes, ceramide synthase (CerS), is responsible in mammals for the reaction of acylation of sphinganine by sphinganine N-acyl transferase. CerS vary in tissue distribution and substrate specificity. CerS generate ceramide with a different acyl chain length (Figure 5). Thus, CerS1, CerS5, and CerS6 are responsible for C18- and C16-long-chain ceramides generation, which adequate contribute to exhibit proapoptotic or antiapoptotic action in endoplasmic reticulum stress (101). C18:0 ceramides, synthesized by CerS1 are crucial for cerebellar development. Furthermore, CerS1 is also the most abundant isoform in skeletal muscle, and therefore changes in its regulation may improve glucose tolerance in high-fat-fed mice (102). CerS2 generates C22-C24-acyl-chain ceramides (34) which influence hepatic, kidney, and lung function (103). Increased expression of CerS2 prevents HeLa cells apoptosis induced by radiation, while expression of CerS5 promotes cellular apoptosis (104). It was shown, that genetically CerS2-deficient mice displayed glucose intolerance even with physiological insulin secretion from the β-cell of the islets of Langerhans. Park et al. reported the lack of receptor phosphorylation in the liver associated with the inability to translocate the receptor to detergent-resistant membranes, which was not found in adipose tissue and skeletal muscle (105). CerS3-derived C24:0 ceramides are essential for skin barrier function (106) (Table 1). Incubation of pancreatic β-cells with high concentrations of palmitate showed an increased expression of CerS4 and induced apoptosis in pancreatic β-cells (107). BMI fat content and hyperglycemia positively correlated with CerS6 expression. CerS6 modulate β-oxidation in brown adipose tissue (BAT) and liver (108). Raichur et al. (109) revealed that CerS6 is a possible therapeutic target for metabolic diseases prevention. Our results indicate that C18:0 and C18:1-derived ceramides play a leading role in fat-induced skeletal muscle insulin resistance. We have shown that intramuscular DAG acyl chain composition in high-fat fed animals was strongly diverse. Only C18:0, C18:1, and C18:2-derived molecular species were elevated, which is related to both the plasma FFA and muscle LCACoA species bioavailability (110). Studies of Raichur and co-workers link C16:0-ceramides with hepatic insulin resistance and suggest the reduction of C16-ceramides level (109). However, C18-derived ceramides were the major species of ceramides stored in skeletal muscle of high-fat fed animals (111), mice with streptozotocin-induced diabetes (112) and in obese, diabetic patients (113).

**Table 1:** Comparison of ceramide synthases (CerS) family.

**Ceramide synthase**	**Tissue expression**	**Acyl-chain lengh**
CerS1	Brain, skeletal muscle, testis	C18
CerS2	Kidney, liver	C20-C26
CerS3	Testis, skin	C22-C26
CerS4	Low level of expression in various tissues	C18-C20
CerS5	Low level of expression in various tissues	C16
CerS6	Low level of expression in various tissues	C14 and C16

*CerS isoforms vary in tissue distribution and substrate preference, thus generating vide range of ceramides with various acyl chain lengths*.

Within 2 decades, knowledge about the etiology of metabolic diseases has increased significantly. The role of lipids in the development of type 2 diabetes is particularly well known, and new strategies for pharmacological intervention are emerging. It is relevant to identify the group of ceramides responsible for the progress of insulin resistance and then synthesis of specific CerS inhibitors.

### Other Approaches

#### Glycerol-3-Phosphate Acyltransferases (GPATs)

**Figure 6:**
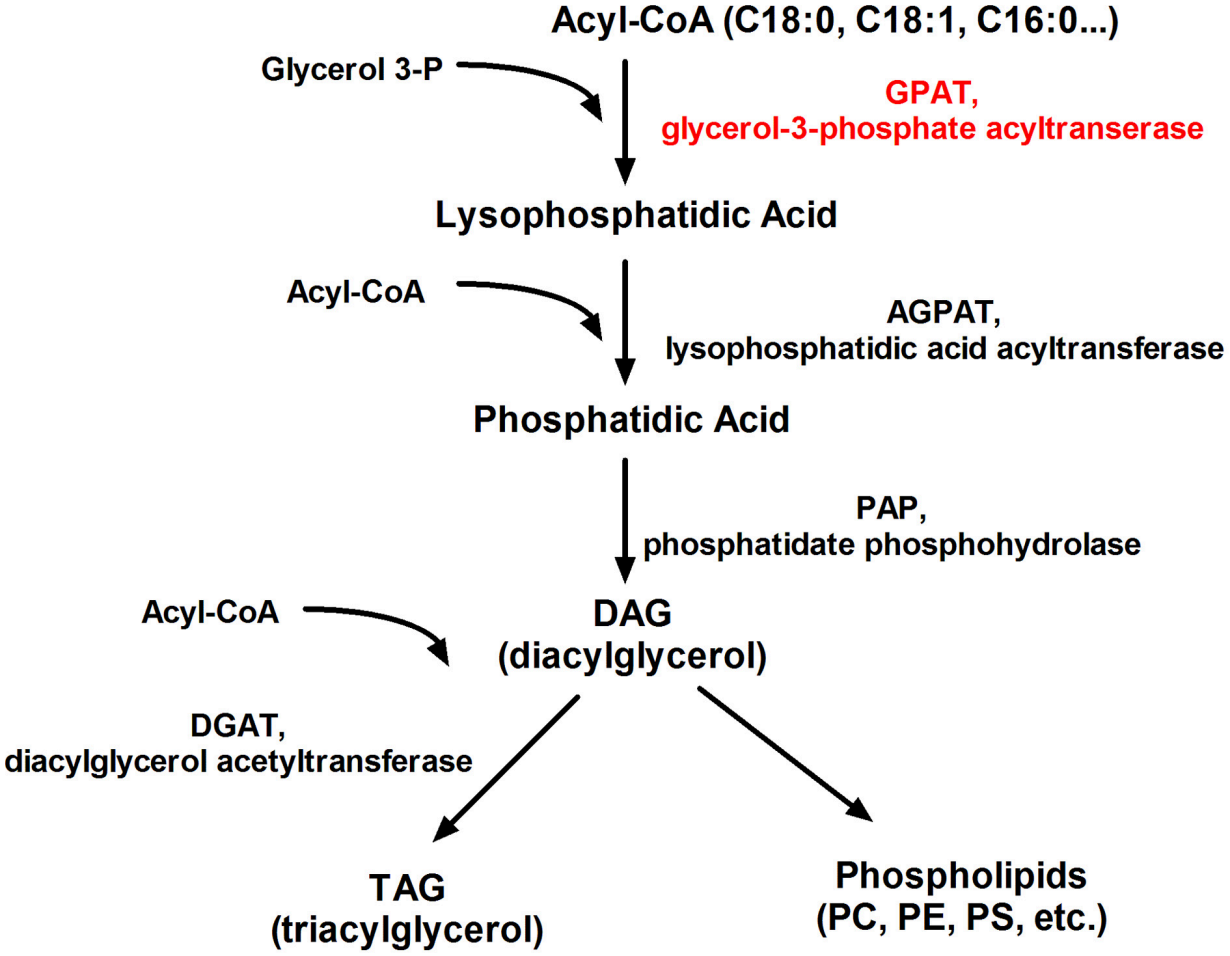
Schematic representation of the glycerolipid metabolism. PC, phosphatidylcholine; PE, phosphatidylethanolamine; PS, phosphatidylserine.

Researchers still looking for new therapeutic options for clinicians who challenge with patients affected by the metabolic syndrome. Four isoforms of GPAT are well described: two mitochondrial (GPAT1, GPAT2) and two microsomal (GPAT3, GPAT4). GPATs are present in the endoplasmic reticulum and the external mitochondrial layer (114), where esterify long-chain fatty-acyl-CoAs (i.e., palmitoyl-CoA by GPAT1) for the biosynthesis of lysophosphatidic acid, a naturally occurring phospholipid which is the first common stage in the production of triglycerides and phospholipids (Figure 6). Thus, GPATs are potential targets for pharmacological treatment of insulin resistance and T2DM (115, 116). Mice with the absence of mtGPAT isoform 1 pose a decreased fat pad, lower body weight, and lower VLDL level (117). The use of recombinant adenoviruses encoding shRNA that knockdown the mtGPAT isoform 1 in the liver of ob/ob mice significantly reduced liver triacylglycerol, diacylglycerol, and FFA content, decreased plasma cholesterol ester levels and reduced high blood sugar (glucose) known as hyperglycemia (118). It was shown that pharmacological GPAT inhibition decreases body weight, obesity, and food intake in mice, simultaneously increasing fatty acid oxidation and preserve against falling energy expenditure caused by hypoxia (119).

## Conclusion

Diet-induced obesity (DIO) is the leading cause of the increasing number of patients diagnosed with metabolic disorders such as type 2 diabetes, cardiovascular disease or atherosclerosis (120). Adiposity is associated with a state of chronic, low-level inflammation accompanied by lipid and glucose levels disorders, which is partially caused by macrophages infiltration into adipose tissue (89). It was suggested that adipose tissue hypertrophy increases the risk of insulin resistance, due to improper deposition of triglycerides in tissues unadapted for safety fat storage (21). In hypertrophied adipose tissue the lipolytic activity is increased what leads to the release of a large amount of FFA that inhibit the insulin action (79, 88). In addition, hypertrophic adipocytes are a source of proinflammatory cytokines that increase insulin resistance (89, 90).

In the last few years, there has been significant progress in understanding the processes of insulin action and molecular defects that give rise to insulin resistance. But many gaps according to the pathophysiology of metabolic disorders still exist in our comprehension. The results of scientific research published over the last 2 decades conducted both on rodents and humans reveal a distinguished role for sphingolipids in insulin resistance in skeletal muscle, liver and fat pad (38, 41, 57, 59, 76, 90). Particular attention has been paid to ceramides as the major suspects in the development of insulin resistance (21). Therefore, changing of ceramides generation may become a desired therapeutic target. First of all, it was shown that increased degradation of ceramides or inhibition of their synthesis ameliorates insulin sensitivity in rodents. For example, insulin signaling improved markedly when ceramide synthesis was inhibited by myriocin and fumonisin B1, but also by increased ceramide degradation by acid ceramidase overexpression. Plasma and tissue ceramide levels also correlate with insulin resistance. Plasma ceramides were higher in overweight children (64), diabetic adults (65), or in non-human primates fed a Western diet (66). Finally, salicylates (92), inflammatory agonists (90), and adiponectin (82) enhance systemic insulin sensitivity.

Researchers have significantly improved the knowledge of the mediators of obesity-related diseases and described a number of various enzymes taking part in different pathways of ceramide metabolism, such as SPT, CerS family or GPATs, which turn to be potential targets to manipulate ceramide generation. Nowadays perspectives of genetic inhibition of enzymes controlling global ceramide synthesis are more often taken into account as an attractive therapeutic goal in the treatment of diseases related to obesity (21, 70, 95, 121). It is worth noting, that ceramides participate in various cellular processes, so risk exists of negative effects of systemic sphingolipid synthesis inhibition. It seems to be extremely important to determine which enzymes in the ceramides pathway participate in the metabolic disease progression. Raichur suggested a reduction in the level of C16-ceramides (109). Nevertheless, regarding ceramide wide biological action, the inhibition of specific ceramide species synthesis could also pose detrimental effects on cellular metabolism. Experimental data suggest that ceramide synthesis and degradation is associated with several crucial cellular responses (apoptosis, cell cycle, and autophagy) (33, 36, 45, 98). García-González and co-workers described an interrelationship between ceramide metabolism and cancer development and progression, highlighting its importance in the modulation of the pivotal processes such as apoptosis, proliferation, migration, invasion, and metastasis (122). Hence, it would be essential to verify whether the ceramide synthesis inhibitors used to prevent insulin resistance would not consequently lead to a carcinogenic effect.

## Author Contributions

ES conceived and drafted the manuscript. AB-Z reviewed the manuscript.

### Conflict of Interest Statement

The authors declare that the research was conducted in the absence of any commercial or financial relationships that could be construed as a potential conflict of interest.

## References

[1] Blachnio-Zabielska A Baranowski M Zabielski P Gorski J. Effect of high fat diet enriched with unsaturated and diet rich in saturated fatty acids on sphingolipid metabolism in rat skeletal muscle. J Cell Physiol. (2010) 225:786–91. 10.1002/jcp.22283 20568228

[2] Gupta A Gupta V. Metabolic syndrome: what are the risks for humans? Biosci Trends. (2010) 4:204–12. 21068471

[3] Chan JC Malik V Jia W Kadowaki T Yajnik CS Yoon KH. Diabetes in Asia: epidemiology, risk factors, and pathophysiology. JAMA. (2009) 301:2129–40. 10.1001/jama.2009.726 19470990

[4] Saltiel AR. New perspectives into the molecular pathogenesis and treatment of type 2 diabetes. Cell. (2001) 104:517–29. 10.1016/S0092-8674(01)00239-2 11239409

[5] Reaven GM. The insulin resistance syndrome: definition and dietary approaches to treatment. Annu Rev Nutr. (2005) 25:391–406. 10.1146/annurev.nutr.24.012003.132155 16011472

[6] Raffel L Goodarzi M Diabetes mellitus. In: Rimoin D, editor. Emery and Rimoin's Principles and Practice of Medical Genetics. New York, NY: Churchill-Livingstone; Elsevier (2013). p. 1–58.

[7] Keller S Lienhard G. Insulin signalling the role of insulin receptor substrate 1. Trends Cell Biol. (1994) 4:115–9. 10.1016/0962-8924(94)90065-5 14731733

[8] Whiteman EL Cho H Birnbaum MJ. Role of Akt/protein kinase B in metabolism. Trends Endocrinol Metab. (2002) 13:444–51. 10.1016/S1043-2760(02)00662-8 12431841

[9] Sano H Kane S Sano E Miinea CP Asara JM Lane WS. Insulin-stimulated phosphorylation of a Rab GTPase-activating protein regulates GLUT4 translocation. J Biol Chem. (2003) 278:14599–602. 10.1074/jbc.C300063200 12637568

[10] Hajduch E Alessi DR Hemmings BA Hundal HS. Constitutive activation of protein kinase B alpha by membrane targeting promotes glucose and system A amino acid transport, protein synthesis, and inactivation of glycogen synthase kinase 3 in L6 muscle cells. Diabetes. (1998) 47:1006–3. 10.2337/diabetes.47.7.1006 9648821

[11] Fulton D Gratton JP McCabe TJ Fontana J Fujio Y Walsh K. Regulation of endothelium-derived nitric oxide production by the protein kinase Akt. Nature. (1999) 399:597–601. 10.1038/21218 10376602 PMC3637917

[12] Summers SA Kao AW Kohn AD Backus GS Roth RA Pessin JE. The role of glycogen synthase kinase 3beta in insulin-stimulated glucose metabolism. J Biol Chem. (1999) 274:17934–40. 10.1074/jbc.274.25.17934 10364240

[13] Takata M Ogawa W Kitamura T Hino Y Kuroda S Kotani K. Requirement for Akt (protein kinase B) in insulin-induced activation of glycogen synthase and phosphorylation of 4E-BP1 (PHAS-1). J Biol Chem. (1999) 274:20611–8. 10.1074/jbc.274.29.20611 10400692

[14] Wang D Sul HS. Insulin stimulation of the fatty acid synthase promoter is mediated by the phosphatidylinositol 3-kinase pathway. Involvement of protein kinase B/Akt. J Biol Chem. (1998) 273:25420–6. 10.1074/jbc.273.39.25420 9738010

[15] Chavez JA Knotts TA Wang LP Li G Dobrowsky RT Florant GL A role for ceramide, but not diacylglycerol, in the antagonism of insulin signal transduction by saturated fatty acids. J Biol Chem. (2003) 278:10297–303. 10.1074/jbc.M212307200 12525490

[16] Ellis BA Poynten A Lowy AJ Furler SM Chisholm DJ Kraegen EW. Long-chain acyl-CoA esters as indicators of lipid metabolism and insulin sensitivity in rat and human muscle. Am J Physiol Endocrinol Metab. (2000) 279:E554–60. 10.1152/ajpendo.2000.279.3.E554 10950822

[17] Schmitz-Peiffer C. Protein kinase C and lipid-induced insulin resistance in skeletal muscle. Ann N Y Acad Sci. (2002) 967:146–57. 10.1111/j.1749-6632.2002.tb04272.x 12079844

[18] Straczkowski M Kowalska I Baranowski M Nikolajuk A Otziomek E Zabielski P. Increased skeletal muscle ceramide level in men at risk of developing type 2 diabetes. Diabetologia. (2007) 50:2366–73. 10.1007/s00125-007-0781-2 17724577

[19] Kang SC Kim BR Lee SY Park TS. Sphingolipid metabolism and obesity-induced inflammation. Front Endocrinol. (2013) 4:67. 10.3389/fendo.2013.00067 23761785 PMC3671289

[20] Perseghin G Scifo P De Cobelli F Pagliato E Battezzati A Arcelloni C. Intramyocellular triglyceride content is a determinant of *in vivo* insulin resistance in humans: a 1H-13C nuclear magnetic resonance spectroscopy assessment in offspring of type 2 diabetic parents. Diabetes. (1999) 48:1600–6. 10.2337/diabetes.48.8.1600 10426379

[21] Chaurasia B Summers SA. Ceramides – lipotoxic inducers of metabolic disorders. Trends Endocrinol Metab. (2015) 26:538–50. 10.1016/j.tem.2015.07.006 26412155

[22] Virkamaki A Korsheninnikova E Seppala-Lindroos A Vehkavaara S Goto T Halavaara J. Intramyocellular lipid is associated with resistance to *in vivo* insulin actions on glucose uptake, antilipolysis, and early insulin signaling pathways in human skeletal muscle. Diabetes. (2001) 50:2337–43. 10.2337/diabetes.50.10.2337 11574417

[23] Sinha R Dufour S Petersen KF LeBon V Enoksson S Ma YZ. Assessment of skeletal muscle triglyceride content by (1)H nuclear magnetic resonance spectroscopy in lean and obese adolescents: relationships to insulin sensitivity, total body fat, and central adiposity. Diabetes. (2002) 51:1022–7. 10.2337/diabetes.51.4.1022 11916921

[24] Thompson AL Cooney GJ. Acyl-CoA inhibition of hexokinase in rat and human skeletal muscle is a potential mechanism of lipid-induced insulin resistance. Diabetes. (2000) 49:1761–5. 10.2337/diabetes.49.11.1761 11078441

[25] Neess D Bek S Engelsby H Gallego SF Faergeman NJ. Long-chain acyl-CoA esters in metabolism and signaling: role of acyl-CoA binding proteins. Prog Lipid Res. (2015) 59:1–25. 10.1016/j.plipres.2015.04.001 25898985

[26] Yu C Chen Y Cline GW Zhang D Zong H Wang Y. Mechanism by which fatty acids inhibit insulin activation of insulin receptor substrate-1 (IRS-1)-associated phosphatidylinositol 3-kinase activity in muscle. J Biol Chem. (2002) 277:50230–6. 10.1074/jbc.M200958200 12006582

[27] Ciapaite J Bakker SJ Diamant M van Eikenhorst G Heine RJ Westerhoff HV. Metabolic control of mitochondrial properties by adenine nucleotide translocator determines palmitoyl-CoA effects. Implications for a mechanism linking obesity and type 2 diabetes. FEBS J. (2006) 273:5288–302. 10.1111/j.1742-4658.2006.05523.x 17059463

[28] Morino K Petersen KF Shulman GI. Molecular mechanisms of insulin resistance in humans and their potential links with mitochondrial dysfunction. Diabetes. (2006) 55:S9–15. 10.2337/db06-S002 17130651 PMC2995546

[29] Randle PJ Garland PB Hales CN Newsholme EA. The glucose fatty-acid cycle. Its role in insulin sensitivity and the metabolic disturbances of diabetes mellitus. Lancet. (1963) 1:785–9. 10.1016/S0140-6736(63)91500-9 13990765

[30] Zhou YP Grill V. Long term exposure to fatty acids and ketones inhibits B-cell functions in human pancreatic islets of Langerhans. J Clin Endocrinol Metab. (1995) 80:1584–90. 10.1210/jc.80.5.1584 7745004

[31] Kelpe CL Moore PC Parazzoli SD Wicksteed B Rhodes CJ Poitout V. Palmitate inhibition of insulin gene expression is mediated at the transcriptional level via ceramide synthesis. J Biol Chem. (2003) 278:30015–21. 10.1074/jbc.M302548200 12771145

[32] Jornayvaz FR Shulman GI. Diacylglycerol activation of protein kinase Cε and hepatic insulin resistance. Cell Metab. (2012) 15:574–84. 10.1016/j.cmet.2012.03.005 22560210 PMC3353749

[33] Galadari S Rahman A Pallichankandy S Galadari A Thayyullathil F. Role of ceramide in diabetes mellitus: evidence and mechanisms. Lipids Health Dis. (2013) 12:98. 10.1186/1476-511X-12-98 23835113 PMC3716967

[34] Holland WL Brozinick JT Wang LP Hawkins ED Sargent KM Liu Y. Inhibition of ceramide synthesis ameliorates glucocorticoid-, saturated-fat-, and obesity-induced insulin resistance. Cell Metab. (2007) 5:167–79. 10.1016/j.cmet.2007.01.002 17339025

[35] Stratford S Hoehn KL Liu F Summers SA. Regulation of insulin action by ceramide: dual mechanisms linking ceramide accumulation to the inhibition of Akt/protein kinase B. J Biol Chem. (2004) 279:36608–15. 10.1074/jbc.M406499200 15220355

[36] Holland WL Summers SA. Sphingolipids, insulin resistance, and metabolic disease: new insights from *in vivo* manipulation of sphingolipid metabolism. Endocr Rev. (2008) 29:381–402. 10.1210/er.2007-0025 18451260 PMC2528849

[37] Itani SI Rudderman NB Schmieder F Boden G Lipid-induced insulin resistance in human muscle is associated with changes in diacylglycerol, protein kinase C and IkB-a. Diabetes. (2002) 51:2005–11. 10.2337/diabetes.51.7.2005 12086926

[38] Nowotny B Zahiragic L Krog D Nowotny PJ Herder C Carstensen M. Mechanisms underlying the onset of oral lipid-induced skeletal muscle insulin resistance in humans. Diabetes. (2013) 62:2240–8. 10.2337/db12-1179 23454694 PMC3712035

[39] Pinel A Rigaudiere JP Laillet B Pouyet C Malpuech-Brugere C Prip-Buus C. N-3PUFA differentially modulate palmitate-induced lipotoxicity through alterations of its metabolism in C2C12 muscle cells. Biochim Biophys Acta. (2016) 1861:12–20. 10.1016/j.bbalip.2015.10.003 26477381

[40] Bikman BT Guan Y Shui G Siddique MM Holland WL Kim JY. Fenretinide prevents lipid-induced insulin resistance by blocking ceramide biosynthesis. J Biol Chem. (2012) 287:17426–37. 10.1074/jbc.M112.359950 22474281 PMC3366851

[41] Goodpaster BH He J Watkins S Kelley DE. Skeletal muscle lipid content and insulin resistance: evidence for a paradox in endurance-trained athletes. J Clin Endocrinol Metab. (2001) 86:5755–61. 10.1210/jc.86.12.5755 11739435

[42] Shoelson SE Lee J Goldfine AB. Inflammation and insulin resistance. J Clin Invest. (2006) 116:1793–801. 10.1172/JCI29069 16823477 PMC1483173

[43] Hotamisligil GS Peraldi P Budavari A Ellis R White MF Spiegelman BM. IRS-1-mediated inhibition of insulin receptor tyrosine kinase activity in TNF-alpha- and obesity-induced insulin resistance. Science. (1996) 271:665–8. 10.1126/science.271.5249.665 8571133

[44] Samad F Hester KD Yang G Hannun YA Bielawski J. Altered adipose and plasma sphingolipid metabolism in obesity: a potential mechanism for cardiovascular and metabolic risk. Diabetes. (2006) 55:2579–87. 10.2337/db06-0330 16936207

[45] Ruvolo PP. Intracellular signal transduction pathways activated by ceramide and its metabolites. Pharmacol Res. (2003) 47:383–92. 10.1016/S1043-6618(03)00050-1 12676512

[46] Schubert KM Scheid MP Duronio V. Ceramide inhibits protein kinase B/Akt by promoting dephosphorylation of serine 473. J Biol Chem. (2000) 275:13330–5. 10.1074/jbc.275.18.13330 10788440

[47] Wang YM Seibenhener ML Vandenplas ML Wooten MW. Atypical PKC zeta is activated by ceramide, resulting in coactivation of NF-kappaB/ JNK kinase and cell survival. J Neurosci Res. (1999) 55:293–302. 10.1002/(SICI)1097-4547(19990201)55:3<293::AID-JNR4>3.3.CO;2-0 10348660

[48] Merrill AH Jr. *De novo* sphingolipid biosynthesis: a necessary, but dangerous, pathway. J Biol Chem. (2002) 277:25843–6. 10.1074/jbc.R200009200 12011104

[49] Hanada K. Serine palmitoyltransferase, a key enzyme of sphingolipid metabolism. Biochim Biophys Acta. (2003) 1632:16–30. 10.1016/S1388-1981(03)00059-3 12782147

[50] Chmitz-Peiffer C Craig DL Bidn TJ Ceramide generation is sufficient to account for the inhibition of the insulin-stimulated PKB pathway in C2C12 skeletal muscle cells pretreated with palmitate. J Biol Chem. (1999) 274:24202–10. 10.1074/jbc.274.34.24202 10446195

[51] Luberto C Hassler DF Signorelli P Okamoto Y Sawai H Boros E. Inhibition of tumor necrosis factor-induced cell death in MCF7 by a novel inhibitor of neutral sphingomyelinase. J Biol Chem. (2002) 277:41128–39. 10.1074/jbc.M206747200 12154098

[52] Lin T Genestier L Pinkoski MJ Castro A Nicholas S Mogil R. Role of acidic sphingomyelinase in Fas/CD95-mediated cell death. J Biol Chem. (2000) 275:8657–63. 10.1074/jbc.275.12.8657 10722706

[53] Cuschieri J Bulger E Billgrin J Garcia I Maier RV. Acid sphingomyelinase is required for lipid Raft TLR4 complex formation. Surg Infect. (2007) 8:91–106. 10.1089/sur.2006.050 17381401

[54] Goldkorn T Balaban N Shannon M Chea V Matsukuma K Gilchrist D. H_2_O_2_ acts on cellular membranes to generate ceramide signaling and initiate apoptosis in tracheobronchial epithelial cells. J Cell Sci. (1998) 111:3209–20. 9763515 10.1242/jcs.111.21.3209

[55] Kolesnick R. The therapeutic potential of modulating the ceramide/sphingomyelin pathway. J Clin Invest. (2002) 110:3–8. 10.1172/JCI16127 12093880 PMC151041

[56] Andrieu-Abadie N Levade T. Sphingomyelin hydrolysis during apoptosis. Biochim Biophys Acta. (2002) 1585:126–34. 10.1016/S1388-1981(02)00332-3 12531545

[57] Amati F Dube JJ Alvarez-Carnero E Edreira MM Chomentowski P Coen PM. Skeletal muscle triglycerides, diacylglycerols, and ceramides in insulin resistance: another paradox in endurance-trained athletes? Diabetes. (2011) 60:2588–97. 10.2337/db10-1221 21873552 PMC3178290

[58] de la MazaMPRodriguezJMHirschSLeivaLBarreraGBunoutD. Skeletal muscle ceramide species in men with abdominal obesity. J Nutr Health Aging. (2015) 19:389–96. 10.1007/s12603-014-0548-725809802

[59] Kolak M Westerbacka J Velagapudi VR Wagsater D Yetukuri L Makkonen J. Adipose tissue inflammation and increased ceramide content characterize subjects with high liver fat content independent of obesity. Diabetes. (2007) 56:1960–8. 10.2337/db07-0111 17620421

[60] Dube JJ Amati F Toledo FG Stefanovic-Racic M Rossi A Coen P. Effects of weight loss and exercise on insulin resistance, and intramyocellular triacylglycerol, diacylglycerol and ceramide. Diabetologia. (2011) 54:1147–56. 10.1007/s00125-011-2065-0 21327867 PMC3804898

[61] Coen PM Menshikova EV Distefano G Zheng D Tanner CJ Standley RA Exercise and weight loss improve muscle mitochondrial respiration, lipid partitioning and insulin sensitivity following gastric bypass surgery. Diabetes. (2015) 64:3737–50. 10.2337/db15-0809 26293505 PMC4613980

[62] Liu L Zhang Y Chen N Shi X Tsang B Yu YH. Upregulation of myocellular DGAT1 augments triglyceride synthesis in skeletal muscle and protects against fat-induced insulin resistance. J Clin Invest. (2007) 117:1679–89. 10.1172/JCI30565 17510710 PMC1866250

[63] Powell DJ Hajduch E Kular G Hundal HS. Ceramide disables 3-phosphoinositide binding to the pleckstrin homology domain of protein kinase B (PKB)/Akt by a PKCzeta-dependent mechanism. Mol Cell Biol. (2003) 23:7794–808. 10.1128/MCB.23.21.7794-7808.2003 14560023 PMC207567

[64] Lopez X Goldfine AB Holland WL Gordillo R Scherer PE. Plasma ceramides are elevated in female children and adolescents with type 2 diabetes. J Pediatr Endocrinol Metab. (2013) 26:995–8. 10.1515/jpem-2012-0407 23612696

[65] Haus JM Kashyap SR Kasumov T Zhang R Kelly KR Defronzo RA. Plasma ceramides are elevated in obese subjects with type 2 diabetes and correlate with the severity of insulin resistance. Diabetes. (2009) 58:337–43. 10.2337/db08-1228 19008343 PMC2628606

[66] Brozinick JT Hawkins E Hoang Bui H Kuo MS Tan B Kievit P. Plasma sphingolipids are biomarkers of metabolic syndrome in non-human primates maintained on a Western-style diet. Int J Obes. (2013) 37:1064–70. 10.1038/ijo.2012.191 23207405 PMC3718866

[67] Warshauer JT Lopez X Gordillo R Hicks J Holland WL Anuwe E. Effect of pioglitazone on plasma ceramides in adults with metabolic syndrome. Diabetes Metab Res Rev. (2015) 31:734–44. 10.1002/dmrr.2662 25959529

[68] Powell DJ Turban S Gray A Hajduch E Hundal HS. Intracellular ceramide synthesis and protein kinase Czeta activation play an essential role in palmitate-induced insulin resistance in rat L6 skeletal muscle cells. Biochem J. (2004) 382:619–29. 10.1042/BJ20040139 15193147 PMC1133819

[69] Manukyan L Ubhayasekera SJ Bergquist J Sargsyan E Bergsten P. Palmitate-induced impairments of β-cell function are linked with generation of specific ceramide species via acylation of sphingosine. Endocrinology. (2015) 156:802–12. 10.1210/en.2014-1467 25535826

[70] Adams JM 2nd Pratipanawatr T Berria R Wang E DeFronzo RA Sullards MC. Ceramide content is increased in skeletal muscle from obese insulin-resistant humans. Diabetes. (2004) 53:25–31. 10.2337/diabetes.53.1.25 14693694

[71] Straczkowski M Kowalska I Nikolajuk A Dzienis-Straczkowska S Kinalska I Baranowski M. Relationship between insulin sensitivity and sphingomyelin signaling pathway in human skeletal muscle. Diabetes. (2004) 53:1215–21. 10.2337/diabetes.53.5.1215 15111489

[72] Hajduch E Balendran A Batty IH Litherland GJ Blair AS Downes CP. Ceramide impairs the insulin-dependent membrane recruitment of protein kinase B leading to a loss in downstream signalling in L6 skeletal muscle cells. Diabetologia. (2001) 44:173–83. 10.1007/s001250051596 11270673

[73] Turban S Hajduch E. Protein kinase C isoforms: mediators of reactive lipid metabolites in the development of insulin resistance. FEBS Lett. (2011) 585:269–74. 10.1016/j.febslet.2010.12.022 21176778

[74] Skovbro M Baranowski M Skov-Jensen C Flint A Dela F Gorski J Human skeletal muscle ceramide content is not a major factor in muscle insulin sensitivity. Diabetologia. (2008) 51:1253–60. 10.1007/s00125-008-1014-z 18458871

[75] Watt MJ Barnett AC Bruce CR Schenk S Horowitz JF Hoy AJ. Regulation of plasma ceramide levels with fatty acid oversupply: evidence that the liver detects and secretes *de novo* synthesised ceramide. Diabetologia. (2012) 55:2741–6. 10.1007/s00125-012-2649-3 22854889 PMC3576922

[76] Blachnio-Zabielska A Zabielski P Baranowski M Gorski J. Effects of Streptozotocin-induced diabetes and elevation on plasma FFA on ceramice metabolizm in rat skeletal muscle. Horm Metab Res. (2010) 42:1–7. 10.1055/s-0029-1238322 19753513

[77] Frayn KN. Adipose tissue as a buffer for daily lipid flux. Diabetologia. (2002) 45:1201–10. 10.1007/s00125-002-0873-y 12242452

[78] Long SD Pekala PH. Lipid mediators of insulin resistance: ceramide signalling down-regulates GLUT4 gene transcription in 3T3-L1 adipocytes. Biochem J. (1996) 319:179–84. 10.1042/bj3190179 8870666 PMC1217752

[79] Kim JI Huh JY Sohn JH Choe SS Lee YS Lim CY. Lipid-overloaded enlarged adipocytes provoke insulin resistance independent of inflammation. Mol Cell Biol. (2015) 35:1686–99. 10.1128/MCB.01321-14 25733684 PMC4405637

[80] Blachnio-Zabielska AU Koutsari C Tchkonia T Jensen MD. Sphingolipid content of human adipose tissue: relationship to adiponectin and insulin resistance. Obesity. (2012) 20:2341–7. 10.1038/oby.2012.126 22677645 PMC3443533

[81] Stern JH Rutkowski JM Scherer PE. Adiponectin, leptin, and fatty acids in the maintenance of metabolic homeostasis through adipose tissue crosstalk. Cell Metab. (2016) 23:770–84. 10.1016/j.cmet.2016.04.011 27166942 PMC4864949

[82] Holland WL Adams AC Brozinick JT Bui HH Miyauchi Y Kusminski CM. An FGF21-adiponectin-ceramide axis controls energy expenditure and insulin action in mice. Cell Metab. (2013) 17:790–7. 10.1016/j.cmet.2013.03.019 23663742 PMC3667496

[83] Holland WL1 Miller RA Wang ZV Sun K Barth BM Bui HH. Receptor-mediated activation of ceramidase activity initiates the pleiotropic actions of adiponectin. Nat Med. (2011) 17:55–63. 10.1038/nm.2277 21186369 PMC3134999

[84] Wellen KE Hotamisligil GS. Inflammation, stress, and diabetes. J Clin Invest. (2005) 115:1111–9. 10.1172/JCI25102 15864338 PMC1087185

[85] Pickup JC Mattock MB Chusney GD Burt D. NIDDM as a disease of the innate immune system: association of acute-phase reactants and interleukin-6 with metabolic syndrome X. Diabetologia. (1997) 40:1286–92. 10.1007/s001250050822 9389420

[86] Uysal KT Wiesbrock SM Hotamisligil GS. Functional analysis of tumor necrosis factor (TNF) receptors in TNF-alpha-mediated insulin resistance in genetic obesity. Endocrinology. (1998) 139:4832–8. 10.1210/en.139.12.4832 9832419

[87] Sartipy P Loskutoff DJ. Monocyte chemoattractant protein 1 in obesity and insulin resistance. Proc Natl Acad Sci U.S.A. (2003) 100:7265–70. 10.1073/pnas.1133870100 12756299 PMC165864

[88] Bray GA Champagne CM. Obesity and the metabolic syndrome: implications for dietetics practitioners. J Am Diet Assoc. (2004) 104:86–9. 10.1016/j.jada.2003.10.041 14702589

[89] Chawla A Nguyen KD Goh YP. Macrophage-mediated inflammation in metabolic disease. Nat Rev Immunol. (2011) 11:738–49. 10.1038/nri3071 21984069 PMC3383854

[90] Kohlgruber A Lynch L. Adipose tissue inflammation in the pathogenesis of type 2 diabetes. Curr Diab Rep. (2015) 15:92. 10.1007/s11892-015-0670-x 26374569

[91] Rajala MW Scherer PE. Minireview: the adipocyte – at the crossroads of energy homeostasis, inflammation, and atherosclerosis. Endocrinology. (2003) 144:3765–73. 10.1210/en.2003-0580 12933646

[92] Goldfine AB Shoelson SE. Therapeutic approaches targeting inflammation for diabetes and associated cardiovascular risk. J Clin Invest. (2017) 127:83–93. 10.1172/JCI88884 28045401 PMC5199685

[93] Yuan M Konstantopoulos N Lee J Hansen L Li ZW Karin M. Reversal of obesity- and diet-induced insulin resistance with salicylates or targeted disruption of Ikkβ. Science. (2001) 293:1673–7. 10.1126/science.1061620 11533494

[94] Hirosumi J Tuncman G Chang L Görgün CZ Uysal KT Maeda K. A central role for JNK in obesity and insulin resistance. Nature. (2002) 420:333–6. 10.1038/nature01137 12447443

[95] Gault CR Obeid LM Hannun YA. An overview of sphingolipid metabolism: from synthesis to breakdown. Adv Exp Med Biol. (2010) 688:1–23. 10.1007/978-1-4419-6741-1_1 20919643 PMC3069696

[96] Ussher JR Koves TR Cadete VJ Zhang L Jaswal JS Swyrd SJ. Inhibition of *de novo* ceramide synthesis reverses diet-induced insulin resistance and enhances whole-body oxygen consumption. Diabetes. (2010) 59:2453–64. 10.2337/db09-1293 20522596 PMC3279530

[97] Xia JY Holland WL Kusminski CM Sun K Sharma AX Pearson MJ. Targeted induction of ceramide degradation leads to improved systemic metabolism and reduced hepatic steatosis. Cell Metab. (2015) 22:266–78. 10.1016/j.cmet.2015.06.007 26190650 PMC4527941

[98] Zheng W Kollmeyer J Symolon H Momin A Munter E Wang E. Ceramides and other bioactive sphingolipid backbones in health and disease: lipidomic analysis, metabolism and roles in membrane structure, dynamics, signaling and autophagy. Biochim Biophys Acta. (2006) 1758:1864–84. 10.1016/j.bbamem.2006.08.009 17052686

[99] Paumen MB Ishida Y Muramatsu M Yamamoto M Honjo T. Inhibition of carnitine palmitoyltransferase I augments sphingolipid synthesis and palmitate-induced apoptosis. J Biol Chem. (1997) 272:3324–9. 10.1074/jbc.272.6.3324 9013572

[100] Chaurasia B Kaddai VA Lancaster GI Henstridge DC Sriram S Galam DL. Adipocyte ceramides regulate subcutaneous adipose browning, inflammation, and metabolism. Cell Metab. (2016) 24:820–34. 10.1016/j.cmet.2016.10.002 27818258

[101] Stiban J Tidhar R Futerman AH. Ceramide synthases: roles in cell physiology and signaling. Adv Exp Med Biol. (2010) 688:60–71. 10.1007/978-1-4419-6741-1_4 20919646

[102] Frangioudakis G Garrard J Raddatz K Nadler JL Mitchell TW Schmitz-Peiffer C. Saturated- and n-6 polyunsaturated-fat diets each induce ceramide accumulation in mouse skeletal muscle: reversal and improvement of glucose tolerance by lipid metabolism inhibitors. Endocrinology. (2010) 151:4187–96. 10.1210/en.2010-0250 20660065 PMC2940499

[103] Laviad EL Albee L Pankova-Kholmyansky I Epstein S Park H Merrill AH. Characterization of ceramide synthase 2: tissue distribution, substrate specificity, and inhibition by sphingosine 1-phosphate. J Biol Chem. (2008) 283:5677–84. 10.1074/jbc.M707386200 18165233

[104] Mesicek J Lee H Feldman T Jiang X Skobeleva A Berdyshev EV. Ceramide synthases 2, 5, and 6 confer distinct roles in radiation-induced apoptosis in HeLa cells. Cell Signal. (2010) 22:1300–7. 10.1016/j.cellsig.2010.04.006 20406683 PMC4348005

[105] Park JW Park WJ Kuperman Y Boura-Halfon S Pewzner-Jung Y Futerman AH. Ablation of very long acyl chain sphingolipids causes hepatic insulin resistance in mice due to altered detergent-resistant membranes. Hepatology. (2013) 57:525–32. 10.1002/hep.26015 22911490

[106] Jennemann R Rabionet M Gorgas K Epstein S Dalpke A Rothermel U. Loss of ceramide synthase 3 causes lethal skin barrier disruption. Hum Mol Genet. (2012) 21:586–608. 10.1093/hmg/ddr494 22038835

[107] Véret J Coant N Berdyshev EV Skobeleva A Therville N Bailbé D. Ceramide synthase 4 and *de novo* production of ceramides with specific N-acyl chain lengths are involved in glucolipotoxicity-induced apoptosis of INS-1 β-cells. Biochem J. (2011) 438:177–89. 10.1042/BJ20101386 21592087

[108] Turpin SM Nicholls HT Willmes DM Mourier A Brodesser S Wunderlich CM. Obesity-induced CerS6-dependent C16:0 ceramide production promotes weight gain and glucose intolerance. Cell Metab. (2014) 20:678–86. 10.1016/j.cmet.2014.08.002 25295788

[109] Raichur S Wang ST Chan PW Li Y Ching J Chaurasia B CerS_2_ haploinsufficiency inhibits β-oxidation and confers susceptibility to diet-induced steatohepatitis and insulin resistance. Cell Metab. (2014) 20:687–95. 10.1016/j.cmet.2014.09.015 25295789

[110] Blachnio-Zabielska AU Chacinska M Vendelbo MH Zabielski P. The crucial role of C18-Cer in fat-induced skeletal muscle insulin resistance. Cell Physiol Biochem. (2016) 40:1207–20. 10.1159/000453174 27960149

[111] Lanza IR Blachnio-Zabielska A Johnson ML Coenen-Schimke JM Jakaitis DR Lebrasseur NK. Influence of fish oil on skeletal muscle mitochondrial energetics and lipid metabolites during high-fat diet. Am J Physiol Endocrinol Metab. (2013) 304:E1391–403. 10.1152/ajpendo.00584.2012 23632634 PMC4116354

[112] Zabielski P Blachnio-Zabielska A Lanza IR Gopala S Manjunatha S Jakaitis DR. Impact of insulin deprivation and treatment on sphingolipid distribution in different muscle subcellular compartments of streptozotocin-diabetic C57Bl/6 mice. Am J Physiol Endocrinol Metab. (2014) 306:E529–42. 10.1152/ajpendo.00610.2012 24368672 PMC3948970

[113] Bergman BC Brozinick JT Strauss A Bacon S Kerege A Bui HH. Muscle sphingolipids during rest and exercise: a C18:0 signature for insulin resistance in humans. Diabetologia. (2016) 59:785–98. 10.1007/s00125-015-3850-y 26739815

[114] Neschen S Morino K Hammond LE Zhang D Liu ZX Romanelli AJ. Prevention of hepatic steatosis and hepatic insulin resistance in mitochondrial acyl-CoA:glycerol-sn-3- phosphate acyltransferase 1 knockout mice. Cell Metab. (2005) 2:55–65. 10.1016/j.cmet.2005.06.006 16054099

[115] Thuresson ER. Inhibition of glycerol-3-phosphate acyltransferase as a potential treatment for insulin resistance and type 2 diabetes. Curr Opin Investig Drugs. (2004) 5:411–418. 15134282

[116] Yu J Loh K Song Z Yang H Lin S. Update on glycerol-3-phosphate acyltransferases: the roles in the development of insulin resistance. Nutr Diabetes. (2018) 8:34. 10.1038/s41387-018-0045-x 29799006 PMC5968029

[117] Hammond LE Gallagher PA Wang S Hiller S Kluckman KD Posey-Marcos EL. Mitochondrial glycerol-3-phosphate acyltransferase-deficient mice have reduced weight and liver triacylglycerol content and altered glycerolipid fatty acid composition. Mol Cell Biol. (2002) 22:8204–14. 10.1128/MCB.22.23.8204-8214.2002 12417724 PMC134068

[118] Xu H Wilcox D Nguyen P Voorbach M Suhar T Morgan SJ. Hepatic knockdown of mitochondrial GPAT1 in ob/ob mice improves metabolic profile. Biochem Biophys Res Commun. (2006) 349:439–48. 10.1016/j.bbrc.2006.08.071 16935266

[119] Kuhajda FP Aja S Tu Y Han WF Medghalchi SM El Meskini R. Pharmacological glycerol-3-phosphate acyltransferase inhibition decreases food intake and adiposity and increases insulin sensitivity in diet-induced obesity. Am J Physiol Regul Integr Comp Physiol. (2011) 301:R116–30. 10.1152/ajpregu.00147.2011 21490364 PMC3129873

[120] Hossain P Kawar B El Nahas M. Obesity and diabetes in the developing world-a growing challenge. N Engl J Med. (2007) 356:213–5. 10.1056/NEJMp068177 17229948

[121] Chavez JA Summers SA. A ceramide-centric view of insulin resistance. Cell Metab. (2012) 15:585–94. 10.1016/j.cmet.2012.04.002 22560211

[122] García-González V Díaz-Villanueva JF Galindo-Hernández O Martínez-Navarro I Hurtado-Ureta G Pérez-Arias AA. Ceramide metabolism balance, a multifaceted factor in critical steps of breast cancer development. Int J Mol Sci. (2018) 19:E2527. 10.3390/ijms19092527 30149660 PMC6163247

